# Oxidative Tea Polyphenols Greatly Inhibit the Absorption of Atenolol

**DOI:** 10.3389/fphar.2016.00192

**Published:** 2016-06-29

**Authors:** Yun Shan, Mengmeng Zhang, Tengfei Wang, Qin Huang, Dan Yin, Zemin Xiang, Xuanjun Wang, Jun Sheng

**Affiliations:** ^1^Key Laboratory of Pu-er Tea Science, Ministry of Education, Yunnan Agricultural UniversityKunming, China; ^2^College of Pu-er Tea, Yunnan Agricultural UniversityKunming, China; ^3^College of Food Science and Technology, Yunnan Agricultural UniversityKunming, China; ^4^College of Life Science, Jilin UniversityChangchun, China; ^5^State Key Laboratory for Conservation and Utilization of Bio-Resources in YunnanKunming, China

**Keywords:** oxidative tea polyphenols, atenolol, drug absorption, interaction, HPLC

## Abstract

Oxidative tea polyphenols (OTPs) is the oxidative polymerization product of epigallocatechin-3-*O*-gallate (EGCG) forms during the process of Pu-er tea fermentation, and possesses absorption property, which may absorbs on drugs thus impact the drug bioavailability when taking medicines with Pu-er tea. Here we demonstrated that OTP inhibited the absorption of atenolol in the intestine, which was determined by testing atenolol levels of plasma via high performance liquid chromatography (HPLC). After administration of atenolol (50 mg/kg), atenolol was absorbed (*T*_max_: 1.867 h) with the half-life (t_1/2_) of 6.663 h in control group; Compared with atenolol group, AUC_0-t_ (h*ng/ml), AUC_0-∞_(h^∗^ng/ml), and *C*_max_ of OTP+atenolol group (OTP 500 mg/kg + atenolol 50 mg/kg) reduced 38.7, 27, and 51%, respectively, the atenolol concentration of plasma was reduced by OTP approximately 43, 49, and 55.5% at 30 min, 1 and 2 h, respectively, (*P* < 0.01). Furthermore, the level of atenolol in feces was higher in the OTP+atenolol group, indicating that the absorption of atenolol in rats was inhibited by OTP. Isothermal titration calorimetry assay identified that EGCG can bind to atenolol and the *in vitro* results showed that OTP absorbed on atenolol and formed precipitate in acid condition, demonstrating a significant positive relationship between atenolol levels and OTP dosage. Taken together, these results suggested that consuming Pu-er tea with atenolol might inhibit atenolol absorption and possible other drugs.

## Introduction

One of the main drug delivery methods is oral administration, and proper absorption is necessary for oral medications to function. The low bioavailability of drugs can be attributed to factors including physiological factors (such as the digestive system and circulatory system) and physical and chemical factors (the solubility of a drug, fat-solubility, dissolution rate, and stability) (Kataoka et al., 2011). The absorption of a single component is often straightforward, but multicomponent drug absorption is inhibited or promoted by interactions. For example, decanoic acid sodium can promote the absorption of berberine in the small intestine (Lv et al., 2010), but administration of oral flavonoids with some medications may result in increased toxicity or a decreased therapeutic effect due to drug interactions (Tang and Stearns, 2001). Further examination of oral drug absorption to improve drug efficacy and to reduce adverse effects is a priority in clinical pharmacy research.

Polyphenols are secondary metabolites of plants belonging to a family of natural organic compounds widely contained in common plant-based foods including vegetables, fruits, cereals, and wine (Spencer et al., 2008). The molecular structure of polyphenols consists of multiple phenolic hydroxyl groups that terminate the propagation of free radical damage by providing active hydrogen to scavenge free radicals, inhibiting lipid peroxidation, inducing complex formation of oxidized transition metal ions, and activating intracellular antioxidant defense systems to reduce the risk of a number of diseases including cardiovascular disease and cancer (Yang et al., 2004; Pandey and Rizvi, 2009; David et al., 2010; Sandhya et al., 2013). As annexing agents widely used in food production, polyphenols usually impact the absorption and metabolism of starch, protein and fat in food. Consumption of polyphenols with certain medications can lead to unexpected interactions. Apple juice could reduce the absorption of a drug but citrus juice could increase the bioavailability of the drug (Jeon et al., 2013). Another type of material containing high levels of polyphenols is tea. Some reports suggest that oral absorption and tissue distribution of metformin are increased after long term use of tea polyphenols, while the absorption and distribution of atenolol were not affected (Hussain and Jaccob, 2013). Research concerning the effects of polyphenols on the absorption of drugs is still in progress.

Tea was considered to reduce the drug effects in ancient Chinese saying, oxidative tea polyphenols (OTPs) is the oxidative polymerization product of EGCG that forms during the process of Pu-er tea fermentation. and possesses strong absorption property to combine with caffeine in Pu-er tea to form a complex, and caffeine can be precipitated under acidic conditions provide a theoretical basis for the phenomenon of the mitigation insomnia effects of fermented Pu-er tea in comparison with other kinds of tea or coffee (Song et al., 2013; Huang et al., 2014). Based on the adsorption property, whether or not OTP affect the absorption of drug when taking medicines with consuming Pu-er tea.

Atenolol is one of the most widely used clinical drugs all over the world, and atenolol is rapidly but incompletely absorbed from the GI tract and demonstrates only approximately 40–50% bioavailability after oral dosing. Approximately 5–15% of atenolol is bound to plasma proteins (Fitzgerald et al., 1978; Scott et al., 1988), and it is often used as pattern drug in drug absorption tests. With atenolol, we investigated the influence of OTP on some drug absorption. The present study was designed to evaluate the effect of OTP on the absorption of a single dose of orally administered atenolol in rats.

## Materials and Methods

### Chemicals and Reagents

Oxidative tea polyphenols was prepared in laboratory as described (Huang et al., 2014): EGCG (the purity is more than 98%) aqueous solution was oxidized with atmosphere oxygen at 37°C under alkaline conditions until the A230 nm/A520 nm (determined by spectrophotometer, HITACHI, U-2910, the optical path length is 1.0 cm, sample concentration is 500 mg L^-1^) equaled 1.40 (the value of the initial tea polyphenols sample is 10.90). Then, the solution was dried by lyophilization.

Atenolol was purchased from Micxy Chemical Co., Ltd (Chengdu, China), EGCG (purity >98%) was purchased from Chengdu Biopurify Phytochemicals Ltd (Chengdu, China). Heparin sodium salt was purchased from Beijing Solarbio Science & Technology Co., Ltd. (Beijing, China). Trichloroacetic acid, NaOH, and KH_2_PO_4_ were purchased from Aladdin (Shanghai, China). Perchloric acid and Hydrochloric acid were purchased from Sigma–Aldrich (Shanghai, China). HPLC-grade methanol and acetonitrile purchased from Tedia Co. Inc. Deionized water was prepared using a purifier (FST-UV-20, Shanghai Fushite instrument equipment Co. Ltd. Shanghai, China). Blank plasma was obtained from Kunming Blood Center (Kunming, China).

### Precipitation of Plasma Protein

Pretreatment of the sample is important for quantitative analysis. A mass of protein is present in plasma and may impact test results. Choosing the appropriate precipitating agent for removal of plasma proteins and test results is crucial. We chose four different types of commonly used protein precipitant mixes with blank plasma at a suitable proportion to eliminate 98% of the protein in plasma: 6% perchloric acid: blank plasma (1:1); 10% trichloroacetic acid: blank plasma (1:1); methanol: blank plasma (3:1); acetonitrile: blank plasma (3:1). After vortex mixing for 30 s, the mixture was centrifuged (CT15RE, Hitachi Koki Co.Ltd, Japan) at approximately 3000 g for 15 min. The supernatant solution was transferred into a disposable 1.5 ml microcentrifuge tube. Next, 20 μl of the final solution was injected into the HPLC system after filtration through a 0.45 μm filter unit (MS^®^ Nylon Syringe Filter and MCE Syinge Filter were purchased from Membrane Solutions, Shanghai, China).

### Isothermal Titration Calorimetry Assay

Isothermal titration calorimetry (ITC) assay was performed on a MicroCal PEAQ-ITC colorimeter (MicroCal, Northampton, MA, USA) to investigate the binding of EGCG to atenolol. Titration calorimetry was performed at 25°C in the assay buffer (PBS, pH = 6.5). Briefly, the sample and syringe cell were filled with atenolol (1.1 mM) and EGCG (13 mM), respectively, which were degassed prior to use. The titrations were conducted using an initial injection of 0.4 μL followed by 18 identical injections of 2 μL with duration of 4 s, and with a 150 s delay between each injection. Curve fitting was performed based on a one-site binding model to determine the equilibrium dissociation constants (KD) using the MicroCal PEAQ-ITC Analysis Software.

### Low-pH Precipitate Method

The atenolol content of the samples was evaluated with the low-pH precipitate method. In brief, the polyphenolic hydroxyl containing components were precipitated under acidic conditions (pH 2) and then dissolved in an alkaline solution. Atenolol bound to polyphenolic hydroxyl containing components can be detected in the solution by HPLC.

A stock solution of atenolol was prepared at a concentration of 10 mg/ml, and a solution of OTP was prepared at a concentration of 100 mg/ml. Various volumes of OTP were pipetted into 1.5 ml microcentrifuge tubes (Nest Biotechnology Co., Ltd. China) (0, 10, 20, 50, 100, 200, 400, 600 μl), and 10 μl of atenolol was added to each tube. Next, 1 M hydrochloric acid was added to each sample solution to adjust the acidity to pH 2; each sample was then diluted to 1 ml with deionized water to yield a final concentration of atenolol 100 μg/ml for each sample. Samples were vortexed for 2 min and centrifuged at 6 000 g at 25°C for 30 min. The precipitate and the supernatant were separated. The precipitate was dissolved in NaOH solution (pH 10). Precipitate solutions were then diluted with deionized water to 1 ml. The atenolol content of the supernatant and the precipitate was determined by HPLC.

### Animals and Study Design

Adult SD rats weighing 200–250 g (8 week old) were obtained from Kunming Medical University (Yunnan, China). The rats were maintained in controlled conditions (12 h light/12 h dark cycle, humidity 50–60%; ambient temperature 24 ± 1°C) and were provided standard laboratory food (ShooBree^®^ Rat Food, Nanjing, China) and water ad libitum. All rat experiments were performed in the animal facility according to the institutional guidelines and were approved by the Institutional Animal Care and Use Committee of the Yunnan Agricultural University.

After acclimatization for a period of one week, the animals were divided into two treatment groups consisting of 10 rats (5 male and 5 female) each: atenolol (50 mg/kg) or OTP (500 mg/kg) + atenolol (50 mg/kg). Rats were food-deprived for 12 h prior to oral administration.

### Plasma and Feces Sample Preparation

Blood samples from the inside canthus were taken at various intervals (5 min, 10 min, 30 min, 1 h, 2 h, 4 h, 6 h, 8 h, 12 h, and 24 h) after drug administration. The blood samples were collected in microcentrifuge tubes with heparin sodium, centrifuged at 6 000 rpm for 10 min, and the resulting plasma was kept frozen at -20°C (DW-40L508, Haier, China) until analysis.

We collected fecal samples at 2, 4, 6, 8, 12, and 24 h after drug administration. The fecal samples were frozen at -80°C (DW-86L626, Haier, China) and dried in a freezer dryer (FD5-6, Sim International Group Co.Ltd. China).

### Analysis of Atenolol in Plasma and Feces

A 200 μl aliquot of rat plasma from the atenolol group was pipetted into a 1.5 ml microcentrifuge tube, and then 200 μl of protein precipitant (6% perchloric acid) was added. After vortex mixing for 30 s, the mixture was centrifuged at approximately 3000 *g* for 15 min. The supernatant solution was transferred into a disposable 1.5 ml microcentrifuge tube. Next, 20 μl of the final solution was injected into the HPLC system after filtration through a 0.45 μm filter unit.

Each rat fecal sample from various time intervals were weighed by an electronic scale and was transferred into a 50 ml centrifuge tube after trituration; a 50-fold volume of feces and mobile phase was pipetted for extraction. After ultrasonic extracting for 1 h, the mixture was centrifuged at approximately 6 000 *g* for 15 min, the extraction liquid was transferred into a disposable 1.5 ml microcentrifuge tube, then 20 μl of the final solution was injected into the HPLC system after filtration through a 0.45 μm filter membrane.

### HPLC-FLD Analysis

Samples were analyzed with an autosampler (G1329B, 1260ALS, Agilent, USA), a fluorescence detector (G1321B, 1260FLD, Agilent, USA), an HPLC pump (G1311B, 1260Quat Pump, Agilent, USA), and a C18 ODS column (ZORBAX SB-C18 4.6 mm*250 mm, 5 microns, Agilent, USA). The analytical method for atenolol was validated using a mobile phase consisting of Acetonitrile-50 mM KH_2_PO_4_ (6:94, v/v pH 4.0) at a flow rate of 1 ml/min, and fluorescence detection was set at an excitation wavelength of 225 nm and an emission wavelength of 300 nm.

### Statistical Analysis

All values were presented as the mean ± standard error of the mean (SEM). Differences within groups were analyzed with repeated measures one-way ANOVA and two-tailed *p* < 0.05 was considered to be statistically significant. All analyses were performed using GraphPad Prism software for Windows (version 5.0 GraphPad Software, Inc., La Jolla, CA, USA).

## Results

### The Content of Atenolol by HPLC-FLD Analysis

Atenolol is a widely used cardio-selective beta-blocker. Quantitative analysis of atenolol in plasma is crucial for the study of pharmacokinetics and guides rational drug use in the clinical setting. Rapid, specific, and sensitive methods for detecting atenolol in plasma are presently available (Kallem et al., 2013).

When comparing the fluorescence detector (FLD) and the UV detector (UVD), sensitivity in the former is far better than in the latter. The resolution of atenolol is affected by several factors; one of these is the type of mobile phase and its components. In this study, the mobile phase consisted of acetonitrile-50 mM KH_2_PO_4_ (6:94, v/v pH 4.0). The use of acetonitrile in the mobile phase is to improve peak shape, shorten retention time and enhance selectivity.

**FIGURE 1:**
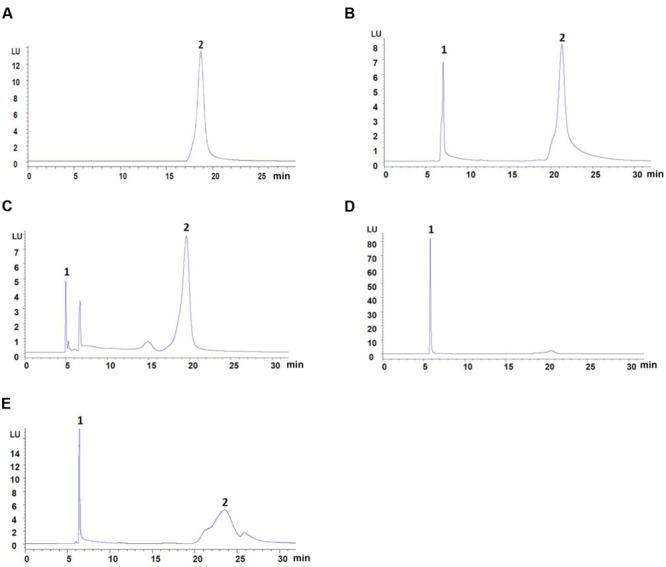
**The content of atenolol by HPLC-FLD analysis. (A)** Mobile phase spiked with 31.25 μg/ml of atenolol. **(B)** Blank plasma spiked with 31.25 μg/ml of atenolol and 6% Perchloric acid. **(C)** Blank plasma spiked with 31.25 μg/ml of atenolol and methanol. **(D)** Blank plasma spiked with 31.25 μg/ml of atenolol and acetonitrile. **(E)** Blank plasma spiked with 44.64 μg/ml of atenolol and 10% Trichloroacetic acid. Peaks: 1, precipitant; 2, atenolol.

Typical atenolol content in blank plasma samples mixed with four different types of protein precipitant is shown in **Figure 1**: 10% trichloroacetic acid group show a low resolution, and acetonitrile group show a low recovery. To evaluate and compare the recovery of all protein precipitant, the linearity of the method was studied for five different concentrations of atenolol. Six percent perchloric acid was chosen as the precipitating agent to add to plasma samples for better separating effect and improved recovery (**Table 1**). The regression curves for atenolol exhibited high linearity *Y* = 11.6^∗^X + 2.5298, *R*^2^ = 0.9999 (*n* = 3).

**Table 1:** The percentage recovery data of atenolol from human plasma with four different kinds of protein precipitant.

Protein precipitant	Average recovery (%)	RSD (%)
10% Trichloroacetic acid	96.73 ± 0.34	0.36
6%Perchloric acid	89.07 ± 0.43	0.48
Acetonitrile	76.06 ± 0.46	0.61
Methanol	69.33 ± 0.70	1.07

*Average recovery are means ± SD of five concentration^∗^100%.*

*RSD (%) = SD/mean^∗^100%.*

### Isothermal Titration Calorimetry Assay

**FIGURE 2:**
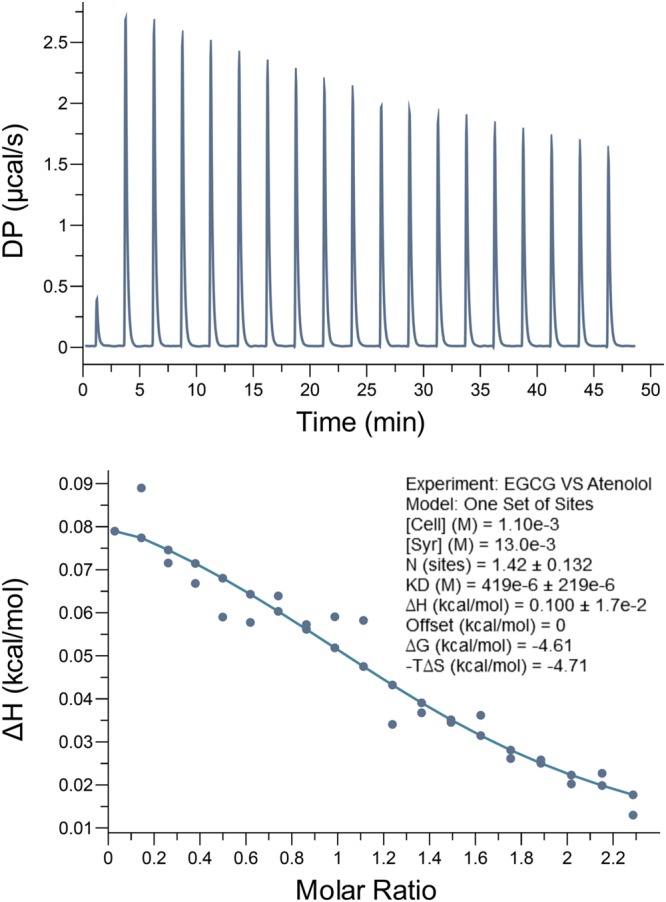
**Epigallocatechin-3-*O*-gallate bind to atenolol *in vitro* by isothermal titration calorimetry (ITC) assay.** The data indicate that the KD is 0.419 ± 0.219 mM for interaction between EGCG and atenolol. Top panel, the titration calorimetry of EGCG with atenolol at room temperature; lower panel, normalized ITC data for titrations versus molar ratio of EGCG with atenolol.

To further identify the interaction of EGCG and atenolol, ITC was performed to assess the binding of EGCG to atenolol (**Figure 2**). In this assay, atenolol was titrated with EGCG at room temperature. The thermodynamics parameters of interaction between EGCG and atenolol can be calculated by fitting the raw ITC data. The parameter values were as follows: N (sites) = 1.42 ± 0.132; ΔH = 0.100 ± 0.017 kcal/mol; ΔG = -4.61 kcal/mol; -TΔS = -4.71 kcal/mol. The equilibrium dissociation constant (KD) was determined after analysis of the normalized ITC curve by the MicroCal PEAQ-ITC Analysis Software. The all data indicated that EGCG can bind to atenolol with a KD of 0.419 ± 0.219 mM and a stoichiometry ratio of 1.42:1.

### The Content of Atenolol by the Low-pH Precipitate Method

**FIGURE 3:**
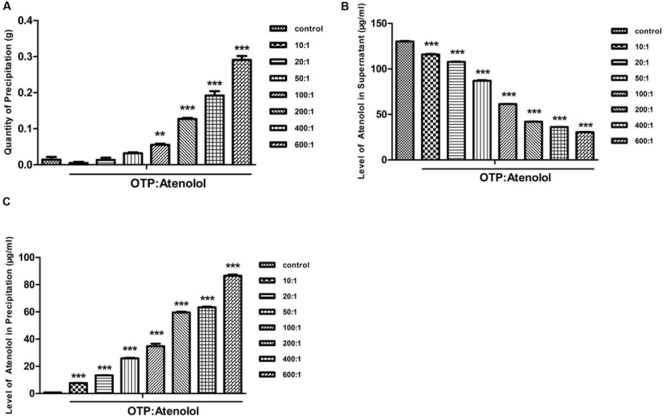
**The content of atenolol by the low-pH precipitate method. (A)** The quantity of precipitation under the condition of pH 2. **(B)** The level of atenolol of supernatant under the condition of pH 2. **(C)** The level of atenolol of re-dissolved solution of precipitation under the condition of pH 10. Data were presented as mean ± SEM (^∗∗^*P* < 0.01; ^∗∗∗^*P* < 0.001) and were analyzed with repeated measure (*n* = 3) one-way ANOVA.

Previous research has shown that atenolol can bind to EGCG. Therefore, in the current study, we set out to observe different ratios of OTP and atenolol (0:1, 10:1, 20:1, 50:1, 100:1, 200:1, 400:1, 600:1). Precipitation formed in low-pH conditions, and the precipitate efficiency of OTP in different quantities was observed (**Figure 3A**).

A high level of atenolol was tested by HPLC-FLD analysis in the supernatant of samples containing OTP (**Figure 3B**). OTP induced a concentration-dependent decrease in atenolol levels. At the ratio of 10:1 (OTP: atenolol) and 100:1, OTP significantly decreased the level of atenolol by 11 and 53% compared to the ratio of 0:1 (*P* < 0.01). These results indicated that a strong positive relationship existed between the dosage of OTP and the precipitate efficiency of OTP.

To further determine whether the reduced content of atenolol is combined with OTP and contained in the precipitate, the precipitate was re-dissolved in pH 10 conditions. A high level of atenolol was tested in the precipitation solution of the sample with a mass of OTP (**Figure 3C**), and the results indicate a negative relationship between atenolol level and the quantity of precipitate in the sample. As the oxidative polymerization product of EGCG, OTP show a strong adsorption with atenolol to correspond with the ITC test result.

### Atenolol Levels of Plasma and Feces

The above results suggest that the atenolol combined with OTP and the mixture precipitated in the environment of low pH. To further characterize the combination of atenolol and OTP in rat gastric juice, atenolol was selected as a control group to investigate whether the absorption of atenolol was affected by the presence of OTP.

**FIGURE 4:**
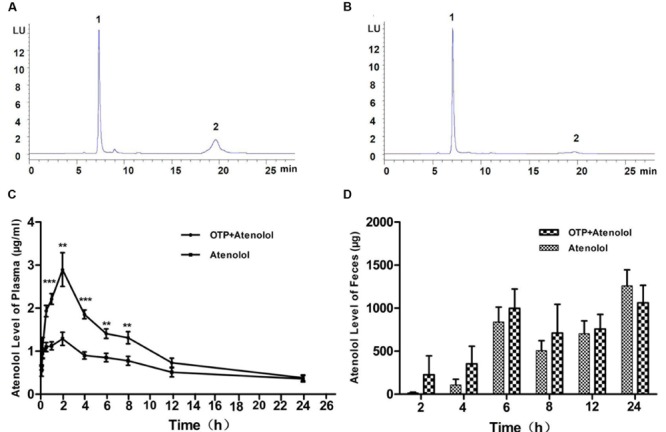
**Atenolol levels of plasma and feces. (A)** The atenolol content of plasma at 1 h after ingestion of atenolol group and **(B)** OTP+atenolol group by HPLC-FLD analysis. The differences in plasma **(C)** and fecal **(D)** level of atenolol at different time point were analyzed with repeated measure (*n* = 10). Data were presented as the mean ± SEM (^∗∗^*P* < 0.01; ^∗∗∗^*P* < 0.001). Peaks: 1, precipitant; 2, atenolol.

After administration of atenolol and OTP for various intervals (5 min, 10 min, 30 min, 1, 2, 4, 6, 8, 12, and 24 h), atenolol levels in plasma were measured by HPLC to examine the absorption of atenolol in rats (**Figures 4A,B**). Atenolol was absorbed (T_max_: 1.867 h) with the half-life (t_1/2_) of 6.663 h, However, compared to the control group (**Table 2**), AUC_0-t_ (h^∗^ng/ml), AUC_0-∞_(h^∗^ng/ml), and C_max_ of OTP+atenolol group reduced 38.7, 27, and 51%, respectively. plasma atenolol levels were significantly reduced by OTP after oral administrations 43, 49, and 55.5% at 30 min, 1 and 2 h, respectively, (*P* < 0.01) (**Figure 4C**). There were no significant differences between the two groups until 12 h after ingestion, and the atenolol levels of plasma demonstrated no significant change in the whole process.

**Table 2:** Pharmacokinetics parameters.

Parameters	Means ± SD	
	OTP+Atenolol	Atenolol
AUC_0-t_ (h^∗^ng/ml)	15.554 ± 2.665	25.378 ± 2.630
AUC_0-∞_(h^∗^ng/ml)	20.996 ± 5.774	28.756 ± 3.803
t_1/2_ (h)	10.898 ± 5.909	6.663 ± 3.337
T_max_ (h)	1.9 ± 2.315	1.867 ± 1.024
C_max_ (μg/l)	1.482 ± 0.170	3.03 ± 0.584

The atenolol levels of plasma in the OTP group were decreased after ingestion, and we speculated that the absorption of atenolol was inhibited in rats. To ascertain the cause of this, feces atenolol levels were examined. The atenolol level in feces from group OTP+atenolol was higher than in the atenolol group (**Figure 4D**). The result indicated that more atenolol was combined with OTP and excreted. In all probability, the absorption of atenolol was inhibited in rats.

## Discussion

Epigallocatechin-3-*O*-gallate is a vital component of tea, which is oxidized to form OTP in the process of Pu-er tea fermentation. ITC assay was performed to assess the binding of EGCG to atenolol show a certain affinity. OTP contain EGCG as the major group (Xu et al., 2016), has higher molecular weight and stronger immune activity, show a stronger affinity with atenolol (Supplementary Figure S1). One possible mechanism is that a large number of active EGCG side-chains present in OTP increase the combining capacity by its affinity. On the other hand, OTP contains a poly phenolic hydroxyl and benzene ring of EGCG with a macromolecular dimensional network structure demonstrating a strong absorbing capacity with small size substances like atenolol by physisorption. Studies on the interaction of OTP and atenolol *in vitro* have illustrated the ability of OTP to affect the solubility of free atenolol in water. Free atenolol is reduced in water when OTP is present under acidic conditions. Another study also found that the ethyl acetate extract fraction of Pu-er tea has almost no monomeric polyphenols, theaflavins, or gallic acid (Jie et al., 2006). To elucidate the mechanism of the OTP-atenolol interaction, we re-dissolved the precipitation in NaOH solution. The data suggested that atenolol was released, and the concentration of atenolol was positively correlated with the precipitation of OTP, demonstrating that the changes in interactions due to pH were reversible.

Atenolol is mainly absorbed by small intestine; one possible mechanism of the reduced level of atenolol in plasma is a potential inhibitory effect on the uptake efficiency of the intestine. Co-administration of atenolol and OTP resulted in the formation of macromolecular complexes that absorbed atenolol in the stomach and sedimented in gastric acid. However, free atenolol cannot separate out under natural conditions. Macromolecular material is not absorbed by the intestine and then excreted, explaining the content of atenolol in feces. Other factors may also be involved. Research suggests that polyphenols inhibit absorption by regulatory intestinal bacteria (Parkar et al., 2013), increase the intestinal mucous layer and inhibit the activity of digestive enzymes and transporters (Schulze et al., 2014; Guri et al., 2015). Atenolol is a hydrophilic compound, with a molecular weight of 266.3. This small size would allow for paracellular absorption and transport through membrane carriers as well (Lennernas et al., 1994). The transport of atenolol across the intestinal epithelium may be mediated by the solute carrier OCT1 (Mimura et al., 2015). OTP may decrease the absorption of atenolol by regulating the expression of drug receptor, but the present study could not confirm this conjecture.

Because of the health benefits of tea, a lot of research more inclined to the positive role of tea, such as combine with anti-cancer drug to promote efficacy and reduce the side effects (Singh et al., 2015; Zaletok et al., 2015). A trend has been observed in recent years: the users and researchers concerning the active effect of food supplements on the health, but the food-drug interactions can result in adverse effects in bioavailability and pharmacokinetics (Margină et al., 2015). Many anti-nutritional compounds in complementary food result in malabsorption (Roos et al., 2013). Edible products also require long-term care. Tea was considered to reduce the drug effects in ancient Chinese saying, mainly because of polyphenol structure are known to chelate metal ions and inhibit the utilization (Kim et al., 2011; Andrews et al., 2014), and exhibit a high affinity with protein via hydrogen bonding and hydrophobic effects (García-Conesa, 2015).

In present study, our finding suggest that OTP from Pu-er tea may reduce the absorption of atenolol in rats by the reason of OTP possessing absorption property, as for the other reaction is not clearly, further experiments should be perform to confirm the findings.

## Conclusion

In conclusion, our results showed that OTP combined with atenolol to become a precipitate in a low-pH environment, and OTP influenced the absorption of atenolol in rats, which may also occur in humans.

## Author Contributions

JS, XW, and ZX conceived and designed the experiments. YS, MZ, TW, QH, and DY performed the experiments and analyzed the data. JS and XW contributed reagents/materials/analysis tools. YS and MZ wrote the manuscript. All authors read and approved the final manuscript.

## Conflict of Interest Statement

The authors declare that the research was conducted in the absence of any commercial or financial relationships that could be construed as a potential conflict of interest.
