# Supplementary material for: Oxidative Tea Polyphenols Greatly Inhibit the Absorption of Atenolol
Source: Front Pharmacol. 2016 Jun 29;7:192. doi: 10.3389/fphar.2016.00192 (PMC4925672; doi:10.3389/fphar.2016.00192)
Supplement: Supplementary file 1 [file Data_Sheet_1.PDF]

Supplementary Material

Oxidative tea polyphenols greatly inhibit the absorption of atenolol

Yun Shan, Mengmeng Zhang, Tengfei Wang, Qin Huang, Dan Yin, Zemin Xiang*, Xuanjun Wang* and Jun Sheng*

***Correspondence:**
E-mail: xiangzmwdx@sohu.com; wangxuanjun@gmail.com; Jun Sheng: shengj@ynau.edu.cn

Structure of Dimeric Catechins Products of EGCG

The preparation process of OTP mentioned in the manuscript was as follows(Huang et al., 2014): epigallocatechin-3-O-gallate (EGCG, the purity is more than 98%) aqueous solution was oxidized. The dimeric catechins (Figure S1) dehydrotheasinensin (A) and diepigallocatechin-3-O-gallate (B) were found from the oxidation products of EGCG (Bailey et al., 1993; Wan et al., 1997). Our first observation of the structure (compound A, B) is that most of the structure of EGCG was exist. Compound A is that the oxidation was occurred B-B'' ring, and the A, C rings and galloyl were not oxidized. Compound B is that oxidative coupling was occurred C-4 and C-8'', and the A, B rings and galloyl were exist. Compared to EGCG monomer, OTP is the oxidative polymerization product of EGCG, which contains EGCG as the major group, has higher molecular weight and stronger immune activity. Research has shown that the mice were immunized with OTP and generated mAbs against EGCG indicated that EGCG side-chain presented in OTP structure(Xu et al., 2016).

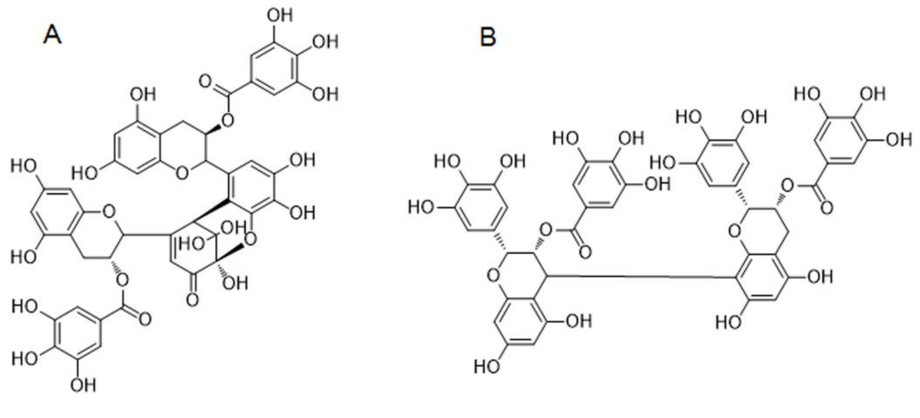

Figure S1. The structure of dimeric catechins products of EGCG : (A) dehydrotheasinensin oxidation was occurred B-B'' ring, and the A, C rings and galloyl were not oxidized. (B) diepigallocatechin-3-O-gallate oxidative coupling was occurred C-4 and C-8'', and the A, B rings and galloyl were exist.

REFERENCES

Bailey, R., Nursten, H., and McDowell, I. (1993). The chemical oxidation of catechins and other phenolics: a study of the formation of black tea pigments. *J Sci Food Agric.* 63(4)**,** 455-464. dio: 10.1002/jsfa.2740630413

Huang, Y.-w., Xu, H.-h., Wang, S.-m., Zhao, Y., Huang, Y.-m., Li, R.-b., et al. (2014). Absorption of caffeine in fermented Pu-er tea is inhibited in mice. *Food Funct. 5(7), 1520-1528. doi: 10.1039/c4fo00051j.*

Wan, X., Nursten, H.E., Cai, Y., Davis, A.L., Wilkins, J.P., and Davies, A.P. (1997). A new type of tea pigment—from the chemical oxidation of epicatechin gallate and isolated from tea. *J Sci Food Agric.* 74(3)**,** 401-408.

Xu, H., Wang, Y., Chen, Y., Zhang, P., Zhao, Y., Huang, Y., et al. (2016). Subcellular Localization of Galloylated Catechins in Tea Plants (Camellia sinensis (L.) O. Kuntze) Assessed via Immunohistochemistry. *Front Plant Sci.* 7**,** 728.
